# Vanadium Decreases Hepcidin mRNA Gene Expression in STZ-Induced Diabetic Rats, Improving the Anemic State

**DOI:** 10.3390/nu13041256

**Published:** 2021-04-11

**Authors:** Cristina Sánchez-González, Lorenzo Rivas-García, Alba Rodríguez-Nogales, Francesca Algieri, Julio Gálvez, Pilar Aranda, María Montes-Bayón, Juan Llopis

**Affiliations:** 1 Biomedical Research Centre (CIBM), Sport and Health Research Centre (IMUDs), Institute of Nutrition and Food Technology, Department of Physiology, University of Granada, E-18071 Granada, Spain; lorenrivas@ugr.es (L.R.-G.); paranda@ugr.es (P.A.); jllopis@ugr.es (J.L.); 2 CIBERehd, Instituto de Investigación Biosanitaria de Granada (ibs.GRANADA), Department of Pharmacology, CIBM, University of Granada, E-18071 Granada, Spain; albarn@ugr.es (A.R.-N.); falgieri@ugr.es (F.A.); jgalvez@ugr.es (J.G.); 3 Department of Physical and Analytical Chemistry, Faculty of Chemistry, University of Oviedo, 33007 Oviedo, Spain; montesmaria@uniovi.es

**Keywords:** vanadium, hepcidin, inflammation, anemia, diabetes, rats

## Abstract

Diabetes is a disease with an inflammatory component that courses with an anemic state. Vanadium (V) is an antidiabetic agent that acts by stimulating insulin signaling. Hepcidin blocks the intestinal absorption of iron and the release of iron from its deposits. We aim to investigate the effect of V on hepcidin mRNA expression and its consequences on the hematological parameters in streptozotocin-induced diabetic Wistar rats. Control healthy rats, diabetic rats, and diabetic rats treated with 1 mgV/day were examined for five weeks. The mineral levels were measured in diet and serum samples. Hepcidin expression was quantified in liver samples. Inflammatory and hematological parameters were determined in serum or whole blood samples. The inflammatory status was higher in diabetic than in control rats, whereas the hematological parameters were lower in the diabetic rats than in the control rats. Hepcidin mRNA expression was significantly lower in the V-treated diabetic rats than in control and untreated diabetic rats. The inflammatory status remained at a similar level as the untreated diabetic group. However, the hematological profile improved after the V-treatment, reaching similar levels to those found in the control group. Serum iron level was higher in V-treated than in untreated diabetic rats. We conclude that V reduces gene expression of hepcidin in diabetic rats, improving the anemic state caused by diabetes.

## 1. Introduction

Vanadium (V) is widely distributed and essential for some living organisms, but its role as a micronutrient for humans, its essentiality, distribution, toxicology, and biological and pharmacological activities are still not fully understood. Although clinical trials for some V compounds have been completed, many aspects remain to be studied, such as their interactions with other elements involved in homeostasis. A growing interest in the pharmacological effects, such as hypoglycemic or insulin-mimetic properties [1,2,3,4], has led V metabolism to become an important area of current research. The following mechanisms of action of V (vanadyl complexes) have been suggested: (a) the in situ formation of peroxovanadates leads to inhibition of PTPases [3,4] in the insulin signaling cascade; (b) vanadyl stimulates cytosolic protein kinases, thus bypassing the insulin receptor altogether; (c) GLUT4 translocation from the intracellular compartment to the plasma membrane. Most likely, the insulin-mimetic activity of V and its possible application as a hypoglycemic agent is associated with the combination of these three effects [1,2,3,4,5]. The administration of organometallic compounds is more effective and less toxic than the administration of the compound in its inorganic form [1,2,3,4,5,6]. Furthermore, some interaction between other trace elements could reduce the toxicity associated with V [7].

Diabetes alters the metabolism of trace metals [8,9]. Diabetes is a disease with an inflammatory component [10,11]. This fact conditions the appearance of a non-ferropenic anemic state in diabetic patients mainly due to two mechanisms: erythropoietin (EPO) hyposecretion and hyporesponsiveness [12,13,14].

According to previous studies, hematological values in non-diabetic rats decreased following a V treatment. Certain studies suggested that V provokes disorders of erythropoiesis and erythrocyte maturation [15,16,17]. However, other studies show that V improves erythropoietic response [18,19].

Hepcidin is a hormone that blocks the intestinal absorption of iron and the release of iron from its deposits. Hepcidin inhibits ferroportin-dependent iron efflux from enterocytes, hepatocytes, and macrophages into the plasma [20,21]. As far as we know, no information about the effect of the treatment with V on hepcidin expression in diabetic rats is available.

Overall, very little is known regarding the effects of V treatment on the metabolism of trace elements, hepcidin expression, and hematological parameters in healthy rats [22]. Nevertheless, no information regarding diabetic rats is available.

Accordingly, the present study aimed to examine whether the treatment of diabetic rats with V causes hematological changes and whether such changes are related to changes in hepcidin expression and the secretion of pro-inflammatory factors, previously demonstrated in healthy rats [22,23]. This study may help clarify the role of V as a micronutrient and its biological activity, potential pharmacological applications, and toxicity.

## 2. Materials and Methods

### 2.1. Animals and Diets

Rats aged 45–48 days at the beginning of the assays were provided by Charles River Laboratories, L’Abresde, France. The weight of the rats used for the experimental section was 190–220 g. The animals were aleatorily distributed into three groups. The control group (C) consisted of 9 rats fed with the AIN93M diet (60 µg V/kg food). The diabetic group (DM) consisted of 10 diabetic rats fed with the semisynthetic diet AIN93M. The diabetic group treated with 1 mg V/day (DMV) consisted of 10 diabetic rats fed with the semisynthetic diet AIN93M and supplemented with 6.22 mg bis(maltolato)oxovanadium(IV) (BMOV)/day added to drinking water. For all the diabetic rats, the diabetes induction by the administration of Streptozotocin (STZ) was performed according to the protocol proposed by Sánchez-González and collaborators [24]. The BMOV preparation and administration, sample collection, and animal conditions have been previously described by our research group [22,25]. All experiments were performed following the Directional Guides Related to Animal Housing and Care (European Council Community, 1986). All procedures were approved by the Ethics Committee on Animal Experimentation of the University of Granada (Reference: CEEA 2011-356).

Samples for the quantitative determination of metals were lyophilized and quantified following the protocol described by our research group [22,25,26].

### 2.2. Determination of Total Metal Concentrations

Diet samples were frozen, freeze-dried, and homogenized to determine total Fe and V content in the diet. The samples were then digested with nitric acid and hydrogen peroxide in a microwave oven. The extracts were collected and diluted for subsequent analysis. Determination of Fe and V in the diet and serum was performed by a collision reaction cell with an ICP-MS (He/H_2_ mode). Calibration curves were prepared using Ga as an internal standard and by diluting stock solutions of 1000 mg L^−1^ in 1% HNO_3_. Five independent determinations of certified reference materials (Seronorm and NIST 8414) were used to validate the method. The percentage of coefficient of variation (CV) for V and Fe was 5.6% and 1.3%, respectively.

### 2.3. Determination of Biochemical and Pro-Inflammatory Parameters

Transferrin (Spinreact Ref 11002134, Girona, Spain), ferritin (Spinreact Ref 1107040DS, Girona, Spain), and C-reactive protein (CRP) (Spinreact Ref 1107001L, Girona, Spain) levels were determined using a BS-200 Chemistry Analyzer (Shenzhen Mindray Bio-Medical Electronics Co., Ltd., Hamburg, Germany). Transferrin saturation (TSAT) was calculated from the ratio (plasma Fe/transferrin) × 71. Leptin, interleukin-1ß (IL-1ß), interleukin-6 (IL-6), and tumor necrosis factor alpha (TNFα) levels were determined following the protocol described by Sánchez-González and co-workers [22].

### 2.4. Measurement of Hematological Parameters

Erythrocyte, leucocyte, lymphocyte and platelet counts, hemoglobin (Hb), and hematocrit (Hct) were determined using a Sysmex KX-21 automatic hematology analyzer (Sysmex, Kobe, Japan). Mean cell hemoglobin (MCH) and mean cell volume (MCV) were calculated from erythrocyte, Hb, and Hct values.

### 2.5. Analysis of Hepcidin Gene Expression in Liver Samples by RT-qPCR

The analysis of hepcidin gene expression in liver were determined following the protocol described by Sánchez-González and collaborators [22].

### 2.6. Ferroportin Expression in HepG2 Cells

HepG2 cells were maintained at the conditions described by Rivas-García and co-workers [7].

Ferroportin expression was evaluated after treating the cells with 500 and 1000 µg/L of BMOV for 48 h. A commercial ELISA kit (Cloud-Clone Corp, Houston, TX, USA) was used according to the manufacturer’s instructions to quantify the protein expression. Prior to the analysis, cells were collected and lysed using RIPA buffer. This type of cells has been previously used by other authors to evaluate the ferroportin expression [27].

### 2.7. Statistical Analysis

Descriptive statistical parameters (means and standard deviations) were obtained for each of the variables studied. The Mann–Whitney U-test for two independent samples and the Kruskal–Wallis test for multiple independent samples were used in the analyses. All the analyses were performed using Statistical Package for Social Science 15.0 (SPSS, Chicago, IL, USA).

## 3. Results

The reasons for the specific dose used and considerations of toxicity problems observed in treated animals have been described in previous publications [28,29]. Some gastrointestinal disorders were found in two animals for the group of rats treated with V; consequently, these animals were removed from the study. These gastrointestinal disorders were also found by other authors [28]. Table 1 describes the food and water intake, diuresis, and glucose level of rats from day 1 to day 35. The DM and DMV groups showed statistically significant differences in all the parameters analyzed compared to the control group.

Serum levels of leptin, CRP, IL-6, IL-1ß, and TNF-α are shown in Table 2. Diabetes decreased serum level of leptin compared with the control group. However, diabetes produced an increase in the level of all the other analyzed pro-inflammatory cytokines. The treatment with BMOV 1 mg V/day in the diabetic rats did not significantly modify leptin level and, overall, this treatment maintained the levels of inflammatory markers similar to those found in the DM group.

Table 3 shows the serum level of V, Fe, transferrin, TSAT, and ferritin. Diabetes increased the serum level of V compared to the control group. The DMV group showed a higher serum level of Fe than the DM group. The magnitude of TSAT showed an upward trend in both DM and DMV groups, although these changes did not reach statistical significance.

**Figure 1:**
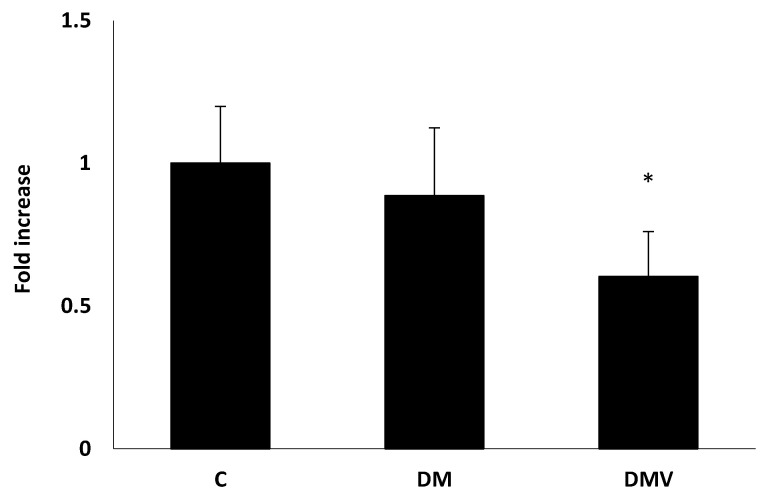
The expression level of hepcidin mRNA in the liver. Values shown are represented as mean ± SD, C (Control rats); DM (diabetic STZ rats); DMV (diabetic STZ rats treated with 1 mg V/day). * Different expression level compared to the C and DM groups C (Mann–Whitney-U test). *p* < 0.05.

The DMV group showed a significantly lower mRNA expression level of hepcidin in the liver than the control group and a ~40% lower than the DM group (*p* < 0.05) (Figure 1).

The count of erythrocytes and lymphocytes and the hemoglobin level was significantly lower in the DM group than in the control group. The DMV group showed a lower count of leucocytes and lymphocytes than the control group but a significantly increased count of erythrocytes and hemoglobin level closer to values found in the control group (Table 4).

**Figure 2:**
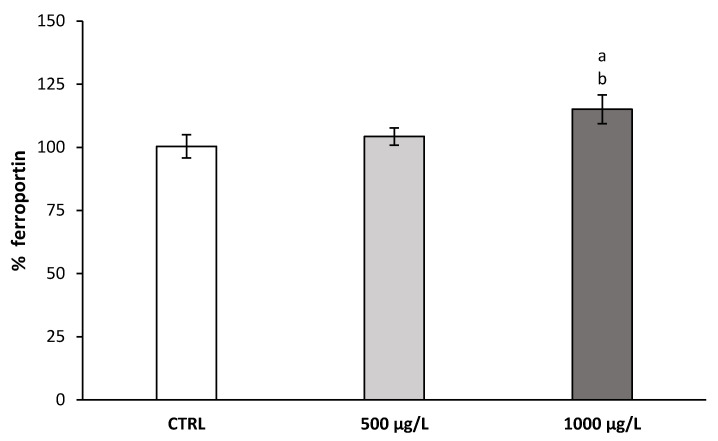
The expression level of ferroportin in HepG2 cells treated with different doses of bis(maltolato)oxovanadium(IV) (BMOV) (500 µg/L and 1000 µg/L). The values shown are represented as mean ± SD, a vs. CTRL; b vs. 500 µg/L. *p* < 0.05.

Furthermore, the changes in ferroportin expression level were evaluated using an *in-vitro* model of hepatic cells. Figure 2 shows that the effect of BMOV supplementation (1000 µg/L for 48 h) promoted a significant increase in protein expression. However, no significant change in ferroportin expression was found when cells were incubated with 500 µg/L BMOV, but a visible upward trend was found compared to the untreated control cells.

## 4. Discussion

### 4.1. Effect of V (IV) Treatment on Leptin and Other Inflammatory Parameters

The DM group showed a lower body weight (b.w. on day 35, C = 296 ± 10 g, DM = 201 ± 15 g, and DMV = 181 ± 3 g) than the other two groups [25] due to the hypercatabolism associated with diabetes. The lower serum leptin level (Table 2) found in DM group was caused mainly by body weight loss [30]. Leptin is an adipokine synthesized mainly by adipose tissue in amounts directly related to adipose tissue mass. This hormone participates in the regulation of appetite (reducing food intake) and energy expenditure [31]. It has been reported that V increases leptin secretion [32] and leptin signal transduction [33]. However, at the present study conditions, treatment with 1 mg V/day (DMV group), although not resulting in significant changes, an upward trend in leptin serum level was found (Table 2) compared with DM rats. A previous study showed no significant differences in hyperglycemia [25]. Therefore, 1 mg V/day showed no changes in the alterations caused by diabetes concerning glycemia or leptin level.

Furthermore, diabetes caused a raise in all the inflammatory markers studied. As stated above, many studies have demonstrated that diabetes courses with a chronic systemic inflammatory status [10,11]. No substantial modification of this situation was found after the V treatment. Inflammatory markers remain similar in diabetic rats after the treatment with BMOV.

### 4.2. Effect of V(IV) Treatment on the mRNA Expression Level of Hepcidin in Liver and Level of Fe, Transferrin, and Ferritin in Serum and Ferroportin Expression in Hepatic Cells

The results showed that the serum level of V in the untreated diabetic (DM) group was higher than in the control group because the animals in the DM group consumed more food than the control group (Table 3), which led to increases in the net quantities of V absorbed (control: 0.23 ± 0.11 μg V/day vs. DM group: 0.64 ± 0.26 μg V/day; *p* < 0.01). The increase in absorbed V is assumed to be responsible for the increase in V serum level for that conditions; V concentration was approximately threefold in the DM group than in the control group (Table 3).

Table 3 and Figure 1 show no changes in hepcidin expression due to diabetes. The expression level of hepcidin was similar in both experimental groups (control and DM groups). Consistent with these results, serum iron level found in the DM group was equal to or slightly higher than in the control group. The DM group showed no significant differences in the level of transferrin, TSAT, and ferritin, although the TSAT and ferritin levels showed a slight upward trend compared to the control group. Available iron not incorporated into erythrocytes would be transported by transferrin and stored in the ferritin deposits.

Albumin and transferrin have been described as responsible for approximately 80–90% of V transport in the blood (as V(IV) or V(V)). Recently, some authors have described, using a lung cell model, that the formation of V(V/IV)-Fe-Transferrin complex slightly decreases the affinity of its binding to TfR1 receptors, which could lead to a decrease in the activity of the V complexes [34]. Because V binds strongly and specifically to the Fe(III) binding sites, the high concentration of V in the plasma may displace Fe from transferrin and facilitate the generation of non-transferrin bound iron (NTBI) complexes, which are readily taken up by macrophages, hepatocytes and other parenchymal cells [21]. Indeed, this situation could facilitate Fe deposits in tissues [35].

Indeed, the present study has demonstrated that V treatment decreased the hepcidin expression in STZ-induced diabetic rats, with the consequent increase in the circulating level of Fe. The finding of a molecule (BMOV) that acts as an inhibitor of the synthesis of hepcidin (intestinal iron absorption inhibitory hormone) increasing the intestinal absorption of iron in an experimental model of inflammatory anemia is of extraordinary importance, considering the complications derived from the oral treatment with the pharmacological iron preparations available in the pharmacopeia. In a previous study, Sánchez-González et al. described that diabetes and the exposure of diabetic rats to V increased the Fe absorbed and retained compared with the control and untreated diabetic rats [35]. These circumstances would facilitate the increase in serum iron level. However, at the present conditions, treatment with 1 mg V/day of DMV group showed no significant differences in the level of glycemia [25] or pro-inflammatory cytokines compared with DM group (Table 2), produced a 40% reduction in the mRNA expression level of hepcidin in the liver compared to the control rats, and a ~30% reduction compared to the DM group (Figure 1). This hormone inhibits ferroportin-dependent iron efflux from enterocytes, hepatocytes, and macrophages into the plasma [21]. An increase of 16% in hepatic ferroportin has been found in rats treated with sodium metavanadate [23], consistent with the results obtained at the present conditions in hepatic cells (Figure 2). The results obtained in the present study showed that the decrease in hepcidin level facilitated an increase in the expression of the ferroportin transporter, thereby allowing a larger efflux of Fe into the plasma (Table 3). This efflux caused an increase in Fe plasma level, that would, in turn, favor the increase in transferrin saturation.

### 4.3. Effect of V(IV) Treatment on Hematological Parameters

The results obtained showed that diabetes decreased the hematological parameters studied (Table 4). In the DM group, the count of erythrocytes and hemoglobin level were significantly lower than in the control group, whereas overall, the other hematological parameters showed a downward trend. This situation reflects the initial phase of the anemia that often accompanies diabetes due to the presence of a chronic inflammatory status [10,11], and in more advanced phases, the development of kidney disease, affecting the production of EPO [36]. EPO deficiency reduces erythropoiesis and shortens erythrocyte life. In the present study, increases in serum level of all the pro-inflammatory cytokines were found (Table 2).

As stated above, diabetes is a disease with an inflammatory component [10,11]. It has been assumed that this fact conditions the appearance of a non-ferropenic anemic state in diabetic patients mainly due to two mechanisms: EPO hyposecretion and hyporesponsiveness. The presence of a high level of pro-inflammatory cytokines causes a decrease in the renal synthesis of EPO. Hyporesponsiveness is defined clinically as a requirement for high doses of EPO to raise blood Hb level in the absence of iron deficiency. It is believed to represent the impaired antiapoptotic action of EPO on proerythroblasts. Possible causes of this EPO hyporesponsiveness include systemic inflammation and microvascular damage in the bone marrow [12,13,14]. In the present study, the effectiveness of erythropoiesis seemed to be reduced due to the inflammatory status that prevails in diabetes. This situation would also be responsible for the slight increase in circulating level of ferritin [37] and in transferrin saturation found in the present study (Table 3). Upward trends in serum level of Fe, TSAT, and ferritin support the hypothesis of the presence of moderate and incipient anemia due to deficient erythropoiesis. The reason why iron level was adequate but not effectively incorporated in the synthesis of new erythrocytes would lie in the presence of inflammatory-induced alterations in erythropoiesis. The chronic inflammatory status inherent to diabetes mellitus is being widely agreed in the scientific community as responsible for alterations in the synthesis and the presence of resistance to EPO [12,13,14]. The linear inverse correlation found between the circulating level of CRP and hemoglobin (*r* = −0.605, *p* < 0.01) supports the previous comments. Moreover, it has been reported that C-reactive protein and IL-6 are associated with hyperglycemia and the development of diabetes [38] and that the presence of a high level of Fe favors inflammatory responses [39]. However, the results obtained in genetic studies seeking to associate IL-6 with type 1 diabetes are inconsistent [40].

**Figure 3:**
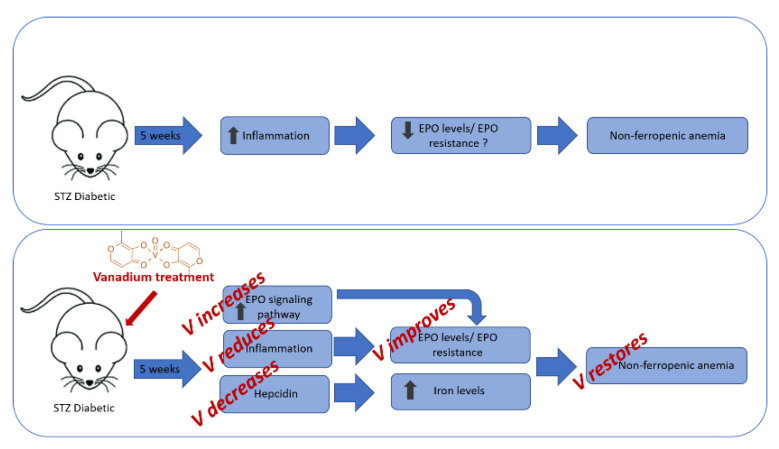
The suggested hypothetical mechanism.

In the present study, the treatment of diabetic rats with 1 mg V/day increased the erythrocyte count and hemoglobin concentration compared to the DM group, achieving values similar to those reported for control rats. In the DMV group, the level of pro-inflammatory cytokines remained similar to those found in the DM group (Table 2), and the level of hepcidin expression was significantly lower (Figure 1), accompanied by an increase in iron serum level (Table 3). Although the inflammatory condition persisted in this V-treated group, and it was, therefore, predicted that EPO effectiveness and the synthesis of marrow erythrocyte precursors remained depressed, data showed that erythropoiesis efficiency improved, increasing both erythrocyte count and the level of hemoglobin. The authors attribute this paradoxical situation to the effect that V has shown on the phosphorylation processes in the intracellular signaling pathways. The kinase stimulating and phosphatase inhibiting effect of V in the intracellular insulin signaling pathway [41,42]. The mechanism of action of EPO and insulin proceeds similarly; in that way, EPO binds directly with the EPO receptor on the surface of erythroid progenitor cells, promoting the activation of some signal transduction pathways. Some changes in the EPO receptor are mediated by the binding of EPO, such as the transphosphorylation associated with Janus kinase 2 (JAK2) molecules, the phosphorylation of tyrosine residues in the cytoplasmic tail of the receptor, and the phosphorylation or the activation of signaling molecules. Phosphorylation of signal transducers and activators of transcription (STAT) 5 transcription factor (TF) produces homodimerization, translocation to the nucleus, and activation of genes for antiapoptotic molecules. Furthermore, phosphorylated phosphatidylinositol 3-kinase (PI-3 kinase) phosphorylates protein kinase B (PKB)/Akt. PKB/Akt (1) phosphorylates and inactivates pro-apoptotic molecules (Bad, caspase 9 or glycogen synthase kinase-3b [GSK-3b]); (2) phosphorylates FOXO TF, inhibiting translocation to the nucleus and activation of target genes (Fas ligand, Bim); and (3) phosphorylates IkB, allowing the release of the transcription factor nuclear factor (NF)-kB that then translocates into the nucleus and activates target genes encoding anti-apoptotic molecules (XIAP, c-IAP2). Additionally, the union of EPO to its receptor triggers some biological actions: it activates Hsp70, that binds to the receptor and inactivates some pro-apoptotic molecules, such as apoptosis protease-activating factor-1 [Apaf-1] and apoptosis-inducing factor [AIF]) [42]. Two studies published in 1994 and 2005 support the hypothesis that V possesses stimulating or mimetic properties of EPO [18,19]. Consequently, V would activate the intracellular signaling pathways of EPO by the same mechanism that activates the intracellular signaling pathway of insulin: inhibiting PTPases or stimulating cytosolic protein kinases. This stimulatory effect of erythropoiesis mediated by its EPO mimetic property in a model of diabetes with an inflammatory component, coupled with its inhibitory effect in hepcidin synthesis, allow V to exhibit anti-anemic potential in diabetes by a double mechanism: the increase in EPO effectiveness and the increase in iron intestinal absorption [35]. These phenomena first point to V as a promising coadjutant in the prevention or anti-anemic therapy in diabetes mellitus, one of the diseases with the highest prevalence and socioeconomic impact, improving the prognosis and quality of life of diabetic patients. The mechanisms of the hypothesis are described in Figure 3.

Moreover, although there is no consensus regarding the effect of V on the number of leukocytes [43], the results obtained from the present study showed that V seems to induce a significant decrease in leukocyte and lymphocyte count. Regarding the platelets count study, despite no significant changes were found in this study, diabetes seems to slightly decrease the platelet count and V tends to limit the effect of diabetes, for that conditions the platelets level was reduced for DM group (679 cells/mm^3^) compared to the control group (513 cells/mm^3^). Regarding the DMV group, the quantity of platelets was intermedium (646 cells/mm^3^). These results are summarized in Table 4.

The limitation of this study is that we have not yet analyzed the exact underlying mechanism of the found effect of V improving the response to EPO. The authors suggest a mechanism similar to that exerted on insulin, which has been demonstrated by other authors in previous studies, acting on different points of the intracellular signaling cascade through phosphorylation processes. However, this aspect is the subject of a new study that we are starting, which will constitute an article by itself. The main strength of the study is that it shows that V seems not only to improve insulin resistance. Nevertheless, its action is effective on other hormones such as EPO, opening a new window for the study of its beneficial effect as a multifunctional compound in other water-soluble hormones resistance, in which the binding to the receptor or its response is limited, perhaps allowing its application in new and numerous diseases.

## 5. Conclusions

The results obtained from this study showed for the first time that V administered at the dose of 1 mg V/day as bis(maltolato)oxovanadium(IV) to diabetic rats reduces the expression level of hepcidin, increases the expression level of ferroportin, and improves the anemic state caused by diabetes. Further studies are needed to determine better the mechanism of action responsible for the found effects, evaluate the effects of these interactions, determine the optimum level of pharmacological intervention, and reduce or prevent side effects.

## Figures and Tables

**Table 1:** Food and water intake, diuresis, and glucose level.

	1st Week				5th Week			
Groups	Food Intake (g/day)	Water Intake (g/day)	Diuresis (mL/day)	Glucose Level (mmol/L)	Food Intake (g/day)	Water Intake (g/day)	Diuresis (mL/day)	Glucose Level (mmol/L)
C	14.1 ± 4.0	17.1 ± 4.7	16.6 ± 4.3	5.0 ± 0.3	15.0 ± 02.0	16.6 ± 4.3	13.0 ± 7.5	4.7 ± 0.3
DM	21.8 ± 6.0 *	156.0 ± 33.0 *	90.4 ± 23.0 *	13.9 ± 2.1 *	30.0 ± 0.9 *	324.0 ± 26.0 *	203.0 ± 43.0 *	14.6 ± 1.2 *
DMV	26.5 ± 3.9 *	167.0 ± 33.0 *	95.7 ± 21.0 *	17.0 ± 2.0 *	22.6 ± 1.2 *	191.0 ± 41.0 *	187.0 ± 46.0 *	19.6 ± 1.7 *

Values shown are expressed as mean ± SD, C (Control rats); DM (diabetic STZ rats); DMV (diabetic STZ rats treated with 1 mg V/day). * Different compared to the C group. *p* < 0.05 [28].

**Table 2:** Serum level of leptin, C-reactive protein (CRP), interleukin-6 (IL-6), interleukin-1β (IL-1β), and tumor necrosis factor alpha (TNF-α) on day 35.

Groups	C	DM	DMV	*p* Test
Leptin (ng/mL)	16 ± 4	2.6 ± 1.0 *	3.2 ± 1.2 *	*p* < 0.001
CRP (mg/L)	2.4 ± 1.5	5.5 ± 1.6 *	5.7 ± 1.4 *	*p* < 0.01
IL-6 (pg/mL)	127 ± 48	193 ± 54 *	197 ± 56 *	*p* < 0.05
IL-1β (pg/mL)	79 ± 23	121 ± 33 *	89 ± 18	NS
TNF-α (ng/mL)	7.7 ± 2.0	11.2 ± 2.2 *	10.1 ± 4.5	*p* < 0.05

Values shown are expressed as mean ± SD, C (Control rats); DM (diabetic STZ rats); DMV (diabetic STZ rats treated with 1 mg V/day). * Different compared to the C group (Mann–Whitney-U test). *p* < 0.05; NS = Not significant (Kruskal–Wallis test).

**Table 3:** Serum level of V, Fe, transferrin, ferritin, TSAT on day 35.

Groups	C	DM	DMV	*p* Test
V (μg/L)	2.4 ± 0.5	6.2 ± 1.2 *	455 ± 96 *^,†^	*p* < 0.001
Fe (mg/L)	1.8 ± 0.1	2.1 ± 0.7	2.7 ± 0.6 *	*p* < 0.01
Transferrin (mg/mL)	6.0 ± 1.4	5.0 ± 1.5	6.9 ± 0.9	NS
TSAT (%)	23 ± 8	30 ± 8	28 ± 5	NS
Ferritin (ng/mL)	211 ± 51	236 ± 18	223 ± 31	NS

Values shown are expressed as mean ± SD, C (Control rats); DM (diabetic STZ rats); DMV (diabetic STZ rats treated with 1 mg V/day). TSAT: transferrin saturation. * Different from group C; ^†^ Different from DM group. (Mann–Whitney-U test). *p* < 0.05; NS = Not significant (Kruskal–Wallis test).

**Table 4:** Hematological parameters on day 35.

Groups	C	DM	DMV	*p* Test
Erythrocytes (×10^6^ cells/mm^3^)	9.1 ± 0.9	8.0 ± 0.3 *	8.8 ± 0.4 ^†^	*p* < 0.05
Hb (g/dL)	16 ± 3	13 ± 1 *	15 ± 1 ^†^	*p* < 0.05
Hct (%)	47 ± 8	44 ± 3	47 ± 3	NS
MCV (fL)	52 ± 5	55 ± 2	53 ± 4	NS
MCH (pg)	18 ± 1	16 ± 1	17 ± 2	NS
Leucocytes (×10^3^ cells/mm^3^)	4.8 ± 1.2	4.1 ± 2.4	2.9 ± 0.5 *	*p* < 0.05
Lymphocytes (×10^3^ cells/mm^3^)	4.3 ± 1.8	2.6 ± 0.9 *	1.9 ± 0.4 *	*p* < 0.01
Platelets (×10^3^ cells/mm^3^)	679 ± 260	513 ± 121	646 ± 122	NS

Values shown are expressed as mean ± SD, C (Control rats); DM (diabetic STZ rats); DMV (diabetic STZ rats treated with 1 mg V/day). * Different compared to the C group. ^†^ Different compared to the DM group (Mann–Whitney-U test). *p* < 0.05; NS = Not significant (Kruskal–Wallis test).
